# Intra-Uterine Injections in the Treatment of Puerperal Septicæmia

**Published:** 1883-08

**Authors:** T. Gaillard Thomas

**Affiliations:** Clinical Professor of Gynæcology in the College of Physicians and Surgeons, New York


INTRA-UTERINE INJECTIONS IN THE TREATMENT OF PUERPERAL SEPTICÆMIA. BY T. GAILLARD THOMAS, M. D., Clinical Professor of Gynæcology in the College of Physicians and Surgeons, New York.

The following case seems to me to illustrate what should be the accepted treatment of puerperal fever, or puerperal septicæmia, at the present day. The case was that of a lady in the higher walks of life whom I was called to see about a month ago, in consultation by her physician, a man of wide experience. She was a primipara, was taken in labor at four o'clock Sunday afternoon, and at nine o'clock in the evening was delivered of a female child, without any difficulty or assistance. Her physician examined the external genitalia carefully, and found no tear whatever. The nurse was instructed to syringe out the vagina carefully the next day with carbolized water, which she did. The first forty-eight hours passed by without any bad symptoms at all, but, on visiting her on Tuesday morning, the physician found a temperature of 101° F., and in the evening it had risen to 102.5°. The next morning, the morning of the fourth day, the temperature was 103°, and the patient began to complain of a very severe pain in the right iliac fossa. There had been no chill. At five o'clock in the afternoon the temperature was 106.5°

in the mouth. The patient's appearance became wild, as of one who was about to have puerperal mania; the skin was hot, and she was crying out with pain, although she had received a good deal of morphine.

Having been called to see the patient, I took the temperature in the mouth myself, and confirmed the record of her physician, that it was 106.5°. The pulse was 145. Making a vaginal examination, I found a bilateral laceration of the cervix uteri extending nearly up the vaginal junction. Probably this extensive laceration partly accounted for the rapidity and the ease of the labor as occurring in a primipara. I urged that the uterus should be washed out with carbolized water at once, but her physician had never seen the method practiced, and was strongly prejudiced against it; he finally consented only because it was apparent that unless something decided was done the patient would soon die. Using the Chamberlain tube and the Davidson syringe, Dr. Jones, and afterward Dr. McCosh, continued to wash out the uterus with carbolized water every four hours during the night, and the next morning the temperature was found to have sunk from 106,5° to 101°; the pulse had fallen from 145 to 120; the patient, who had been given opium quite freely during the night, declared that she was very much relieved. Indeed, the relief had been so extraordinary that they began to believe that the danger was not real at all; and that exceptional circumstance had occurred, and that there was no septicæmia. The uterus was now washed out at longer intervals, but ato nce the temperature went up to 102°, 103°‘ 104° and 105°, and the patient again began to look maniacal. The uterus was now washed out every three hours, opium was freely administered, ten grains of quinine were administered every eight hours, ice water was passed through a coil of rubber tubing placed over the abdomen; and as long as this treatment was kept up the temperature did not rise above 101° or 102°, but as soon as they ceased to wash out the uterus the temperature at once rose to 104°, and at times to 105°. This fact was proved by repeated trials.

After this treatment had been continued for ten days, a physician remaining with the patient day and night, giving the injections every three hours, and thirty grains of quinine during the course of the day, it was believed to be time to stop it; but in less than twenty-four hours the temperature again arose to 105°. I mention the amount of quinine which was being taken particularly, so as to prove positively that there was nothing of a malarial character in the case at all. On the sixteenth day after delivery, the tenth day after the commencement of the high temperature, the intervals between the uterine injections were extended from three hours to four, then to five, six and seven hours, and finally they were discontinued altogether, and at the same time the administration of quinine was given up and the coiled tubing was taken off. Opium was continued in small doses for a while longer, and the patient recovered entirely.

I wish to contrast this case with another which I saw just before—that of a woman who had been recently delivered of her third child. When I was called to see the patient the temperature was 106°; she had been taken with violent pain in one iliac fossa, and had been put five days before pretty profoundly under the influence of opium, and a blister had been applied over the whole of the abdomen. Large doses of quinine had likewise been administered. When I saw the patient, the use of intra-uterine injections was begun at once, but the patient lived only twenty-four hours and died in a state of coma.

It seems to me that the time has arrived when puerperal septicæmia should be treated upon just as simple a plan as septicæmia of any other kind is, namely, by washing with some antiseptic fluid the surface where the disease originates—some fluid which will remove the poisonous material which is being absorbed, and also, so far as possible, neutralize its poisonous qualities. In brief, I would say that puerperal septicæmia, with our present light on the subject, should be treated in the following manner: First, wash out the uterine cavity completely with some antiseptic fluid; second, quiet all pain by opium; third, get the peculiar influence of quinine upon the nervous system; and, fourth, keep temperature, at all hazards, at or below 100° by the methods which we now possess. Three years ago, at the American Gynæcological Society, which met in Baltimore, I took the ground which I take to day regarding this subject, and only one gentleman in the entire society supported my view. Every other member who spoke referred to the dangers of introducing air into the uterine sinuses during the injection, etc. But I believe that the dangers attending the use of the injections are counterbalanced by the benefits to be derived. I do not think there is the leas probability that air will be introduced if a tube of a large size—as large as the finger—is used. But when a catheter is employed there is some danger in inserting it into a sinus and introducing air and fluid together directly into the vessels.—*New York Med Jour.*

